# Supplementary material for: Polyphyletic ancestry of expanding Patagonian Chinook salmon populations
Source: Sci Rep. 2017 Oct 30;7:14338. doi: 10.1038/s41598-017-14465-y (PMC5662728; doi:10.1038/s41598-017-14465-y)

Supplementary Information

Polyphyletic ancestry of expanding Patagonian Chinook salmon populations

Cristian Correa[1, 2,*], and Paul Moran[3]

[1] Facultad de Ciencias Forestales y Recursos Naturales, Instituto de Conservación Biodiversidad y Territorio, Universidad Austral de Chile, Casilla 567, Valdivia, Chile.

[2] Facultad de Ciencias, Instituto de Ciencias Marinas y Limnológicas, Universidad Austral de Chile, Casilla 567, Valdivia, Chile.

[3] Conservation Biology Division, Northwest Fisheries Science Center, Seattle, Washington, United States of America.

* Author of correspondence, E-mail: cristiancorrea@gmail.com

Table S1: Chinook salmon deliberate and accidental releases in Latin America (modified from Correa and Gross 2008).

**Country year(s)**<br>**Basin, Latitude**<br>**River stocked**	**Number of Individuals Released**	**Ontogenetic Stage**	**Stock Origin**	**Adult Returns**	**Comments**
Mexico 1891-1900<br>?	50,000[1]	?	USA[2], Sacramento River (?)[1]	No[1,2]	"From [1872-1930] the [US] Bureau of Fisheries, with benevolent intent, supplied over 100 million eggs of Pacific salmon (Chinook) to people in other countries, with the idea of establishing new salmon runs there—a considerable attempt to bring in the New World to right the Rest."[3]
Mexico 1901-1910<br>?	50,000[1]	?	USA[2], Sacramento River (?)[1]	No[1,2]	
Nicaragua 1901-1910<br>?	20,000[1]	?	USA[2], Sacramento River (?)[1]	No[1,2]	
Argentina 1906<br>Santa Cruz, 50°S[4]<br>Gallegos, 52°S[4]	300,000[4]	?	USA, Sacramento River (?)[4]	No (?)[5]	
Argentina 1908<br>Chico, 50°S[6]<br>Santa Cruz, 50°S[6]	300,000[6]	?	USA, Sacramento River (?)[6]	No (?)[5]	
Argentina 1909<br>Chico, 50°S[6]<br>Santa Cruz, 50°S[6]	200,000[6]	?	USA, Sacramento River (?)[6]	No (?)[5]	
Argentina 1910<br>(Rivers of Santa Cruz Province[6])	200,000[6]	?	USA, Sacramento River (?)[6]	No (?)[5]	
Argentina 1901-1910[1]<br>?	1,058,000[1]	?	USA[2], Sacramento River (?)[1]	No?[2,5]	The last Argentinean entry (1901-1910) might include the previous four.

Brazil, 1958<br>Jaquarí, 30°S<br>Río Caí[7]<br>RíoTainhos[7]<br>Río dos Antos[7] (*sic*)	400,000[7]	Fertilized eggs[7]	USA, American River, California[7]	No (?)[7]	Although there were no reports of salmon returning to the Río Jaquarí, large fish of a species unknown to local residents were seen leaping falls in the Río Uruguay in 1962[7].
Chile, 1924<br>Imperial, 39°S<br>Río Cautin8[, 9]<br>Maullin,42°S<br>Río Maullín[8,9]<br>Cochamó, 42°S<br>Río Cochamó[9]<br>Puelo,42°S<br>Río Puelo[8,9]	200,000[9,10]<br>(little less than 50% died during transport[9])	Fingerlings<br>(4mo[9])	USA, Sacramento River; USA, McCloud River Hatchery (?)[10]	No (?)[8,9,10]	The U.S. government presented the government of Chile with 200,000[10,9] fertilized ChS eggs. The embryos arrived at a recently built hatchery in Rio Blanco (near Santiago) just prior to hatching[8,9]. After four months, fingerlings were transported by rail and released[9].
Chile 1970, 1971<br>Bueno, 40°S<br>Río Chirri[11]	50,000 (1970)[11]<br>270,150 (1971)[11]	Subyearlings<br>(8mo, 15g, 12cm in 1970; 5mo, 5.5-17g, 6-12cm in 1971)[11]	USA, Green River Hatchery[11] (Cowlitz River, lower Columbia River, Washington).	?	Agriculture and Livestock Service of the Government of Chile and the U.S. Peace Corps. First shipment by plane[11,12]. Eggs were received at Lautaro Hatchery on December; stockings took place 36 (Sep 1970) or 23 weeks later (May 1971)[11].
Chile, 1978<br>Coastal, 42°S<br>Chiloé Island,<br>Curaco de Vélez[10,13,14,15]	120,000<br>(late 1978)[13,14,16]<br>170,000[10,15]	Smolts (1+; 70g)[13,14]	USA, Cowlitz River spring-run (lower Columbia River, Washington)[16]	Yes[13,14,16]	Domsea Pesquera Chile Ltd. (Union Carbide Corporation, USA), began salmon ocean-ranching experimentation[10,14,15]. In 1979, 334 returning jacks and 2 females were trapped[13,14]. In the period 1979-1982, 1050 returnees of this release were recorded[16].

Chile, 1979<br>Coastal, 42°S<br>Chiloé Island,<br>Curaco de Vélez[14]	190,000[13,14]	Smolts (1+)[13,14]	Idem[16]	Yes[13,14,16]	Domsea Pesquera Chile Ltd.[13,14]. In the period 1980-1982, 228 returnees of this release were recorded[16].
Chile, 1980-1981<br>Same location[16]	90,000 (late 1980, early 1981)[16]	Smolts (1+)[16]	USA, Bonneville Hatchery fall-run (Columbia River, Washington)[16]	Yes[16]	In the period 1981-1982, 260 returnees of this release were recorded[16].
Chile, 1982<br>Same location[16]	3000[16]<br>(early 1982)	Smolts (1+)[16]	USA, University of Washington's Hatchery fall-run[16]	?	In 1981, Domsea Pesquera Chile Ltd. was sold to Fundación Chile (private, non-profit), and renamed Salmones Antártica Ltd.[10,15]. Stocking continued at this location at least during the first year of the new administration.
Chile, 1982<br>Same location[16]	>1.000.000 fish were being raised[16], but their fate remains unknown to us.	Ova & subyearlings[16]	USA, University of Washington's Hatchery fall-run[16] and Bonneville Hatchery fall-run[16]; Chile, progeny from local returnees[10,15,16]	?	Through October 1982, 1538 adults had returned to the hatchery from previous brood years. Returnee's progeny (F2) was being raised at the facility along with fry from two additional importations[16]. However, we found no posterior records of fish release at this location.
Chile, 1982<br>Coastal, 54°S<br>Río Santa María[15,16,17]	200,000[16,17]	Fry[16]	USA, University of Washington's Hatchery fall-run[15,16,18]	?[15,18]<br>Yes19	Fundación Chile through Salmones Antártica Ltd. launched new facility in the Magellan region subsequently destroyed by storm, and abandoned[15]. Jacks seen returning in 1983[19].
Chile, 1983<br>Prat, 51°S<br>Río Prat[20]	5,000[20]	Smolts (1+)[20]	USA[20], University of Washington's Hatchery fall-run[18,21] (?); Chile, progeny from returnees at Curaco de Vélez and Astilleros, Chiloé[20,21].	Yes<br>(~2.3%)[20]	Fundación Chile through Salmones Antártica Ltd. launched another facility in the Magellan region with successfully returning spawners[15,17].

Chile,1987<br>Same location[20]	294,967[20]<br>(USA origin)<br>40,042[20]<br>(Chilean origin)	Smolts (1+)[20]	USA[20], seemingly University of Washington's[18];<br>Chile, progeny from returnees[20] at Río Prat (local), Curaco de Vélez, and Astilleros[22]	Yes<br>(~0.07% until 1989)[20]	Continuation of the above enterprise. In 1998 Fundación Chile and Salmones Antártica created Salmotec S.A.[23].
Chile, 1989,1990, 1993<br>Bueno 40°S<br>Estero Huillín[24]	? (1989-1990)<br>3347 (Jan 1993)[24]	Smolts (0+)	? (1989-1990);<br>Chile, progeny from 1992 returning adults (38 females + 12 males)[24]	Yes, at least from 1989-1990 stockings[24]	Universidad de Los Lagos' experimentation at Piscicultura Experimental Lago Ranco[24].
Chile, 1987-2000<br>Coast, 39-45°S<br>Inner seas[25]	100,000[26]	Mostly subadults[27]	USA, Washington Sate[28,29]<br>USA, Oregon State[30]<br>Canada, Vancouver Island[31]<br>New Zealand[32]<br>USA, Alaska[33] (?)<br>Australia[34] (?)	Yes[35]	Chinook stocks were imported primarily to the Lakes District Region for commercial net pen rearing. Last recorded importation in 2000.

Notes and references: The actual number of individuals released may be less than the figure reported due to mortality during transport and handling; pre-release mortality was accounted for whenever possible. Approximate latitude is given at the river mouth. ? = unreported, likely stock origin, or lack of adults return assessment; [1] Davidson and Hutchinson (1938); [2] Welcomme (1988); [3] Elton (1958); [4] Tulian (1908) in Ciancio et al. (2005); [5] Marini (1936) in Davidson and Hutchinson (1938); [6] Marini and Mastrarrigo (1963) in Ciancio et al. (2005); [7] Joyner (1980); [8] Golusda (1927); [9] Barros (1931); [10] Fundación Chile (1990); [11] Snyder (1971); [12] Ellis and Salo (1969) in Basulto (2003); [13] Lindbergh et al. (1981); [14] Lindbergh (1982); [15] Méndez and Munita (1989); [16] Lindbergh and Brown (1982); [17] Basulto (2003); [18] Donaldson and Joyner (1983); [19] Manuel Barros personal communication (2008) in Aedo (2011). At the time, M. Barros worked for Fundación Chile.; [20] Salmotec Ltd. in Sakai (1989); [21] Cristian Jélvez personal communication (2005) in Aedo (2011). C. Jélvez worked for Fundación Chile (1982).; [22] Fredy Carrasco personal communication (2005) in Aedo (2011). F. Carrasco worked for Fundación Chile (1986).; [23] United Nations (2006); [24] Del Real (1993). Aedo (2011) mentioned other stocking locations (Río Contaco and Río Maicolpué) by Universidad de Los Lagos, but we found no further records of these releases.; [25] Primarily marine aquaculture concessions in the Lake District region.; [26] Rough estimate of number of sub-adult Chinook salmon escapees (see main text).; [27] Mostly 1+ year class and older since most escapes were from marine net-pens (Soto et al. 2001).; [28] Follow fragmentary records of ova imported (OI) by the Chilean aquaculture industry in 1987-2000 (Aedo 2011). Some information of suppliers was available for 60% of the imports; we report specific lineages and origins of livestock whenever possible, and ova suppliers and/or geographic origin of shipments otherwise. Additional potential sources of the unaccounted imports were identified from import permits (OP) issued by the Chilean National Fisheries Service (SERNAPESCA), although it remains unclear if these planed importations ever materialized. Sources listed in decreasing order of importance (Aedo 2011); [29] OI: Columbia River. OP: Fish Pro Inc. and University of Washington; [30] OI: Springfield. OP: Aqua Food, Aquafoods, and Aqua Seed Corp.; [31] OI: Koksilah River. OP: Sea Spring Salmon Farms Ltd., Hardy Sea Farms, Hadfield Consultants Inc., Hatfield International SA., Fishpro, and Aqua Seed; [32] OI: Sanford Waitaki Salmon Hatchery (Kaitan Gata). OP: Big Glory Bay Hatchery, and Kaitan Gata Hatchery and Sanford Waitaki Salmon Hatchery (Stewart Island).; [33] OP: Sitka; [34] OP: Tasmania; [35] This study.

References

Aedo, E. 2011. Información sobre siembras de salmónidos en el ambiente natural e incidencia de escapes desde centros de cultivo en la Región de Aysén, referenciada geográficamente. *In* Evaluación cuantitativa del estado trofico de salmonidos de vida libre en el fiordo Aysén, XI región. Informe final proyecto FIP200830. *Edited by* E. Niklitschek and P. Toledo. Universidad Austral de Chile - Subsecretaría de Pesca, Puerto Montt. pp. 120–135.

Barros, R. 1931. Introducción de un nuevo salmón en Chile. Revista Chilena de Historia Natural **35**: 57–62.

Basulto, S. 2003. El largo viaje de los salmones. Una crónica olvidada. Propagación y cultivo de especies acuáticas en Chile. Maval Ltda., Santiago.

Ciancio, J.E., Pascual, M.A., Lancelotti, J., Rossi, C.M.R., and Botto, F. 2005. Natural colonization and establishment of a chinook salmon, *Oncorhynchus tshawytscha*, population in the Santa Cruz River, an Atlantic basin of Patagonia. Environmental Biology of Fishes **74**: 219–227.

Correa, C., and Gross, M.R. 2008. Chinook salmon invade southern South America. Biological Invasions **10**: 615–639. doi: 10.1007/s10530-007-9157-2.

Davidson, F.A., and Hutchinson, S.J. 1938. The geographic distribution and environmental limitations of the Pacific salmon (genus Onchorhynchus). Bulletin of the Bureau of Fisheries **48**: 667–692.

Donaldson, L.R., and Joyner, T. 1983. The salmonid fishes as a natural livestock. Scientific American **249**: 50–58.

Elton, C.S. 1958. The ecology of invasions by animals and plants. University of Chicago Press.

Fundación Chile. 1990. El libro del salmon. Fundación Chile, Santiago.

Golusda, P. 1927. Aclimatación y cultivo de especies salmonídeas en Chile. Boletin de la Sociedad de Biología de Concepción **1(1 y 2)**: 80–100.

Joyner, T. 1980. Salmon ranching in South America. *In* Salmon ranching. *Edited by* T. John E. Academic Press Inc., London, England.

Lindbergh, J., Noble, R., and Blackburn, K. 1981. First returns of Pacific salmon to Chile. C.M. 1981/F:27 International Council for The Exploration of the Sea.

Lindbergh, J.M. 1982. A successful transplant of Pacific salmon to Chile. Proceedings of the Gulf and Caribbean Fisheries Institute **34**: 81–87.

Lindbergh, J.M., and Brown, P. 1982. Continuing experiments on salmon ocean ranching in southern Chile. C.M. 1982/M:21 International Council for The Exploration of the Sea. Available from http://www.ices.dk/sites/pub/CM%20Doccuments/1982/M/1982_M21.pdf [accessed 28 June 2016].

Méndez, R., and Munita, C. 1989. La salmonicultura en Chile. *In* Primera edición. Fundación Chile, Santiago.

Del Real, A. 1993. Antecedentes sobre el cultivo de salmón Chinook (*Oncorhynchus tshawystcha* W.) en la fase de agua dulce proveniente de reproductores retornantes en la piscicultura experimental Lago Rupanco. Seminario (Ingeniería de Ejecución en Acuicultura), Universidad de Los Lagos, Departamento de Acuicultura y Recursos Acuaticos, Osorno, Chile.

Sakai, M. 1989. Final report of aquaculture project in Chile. Japan International Cooperation Agency (JICA).

Snyder, B.P. 1971. Supplemental report on inland fresh water resources of central Chile.

Soto, D., Jara, F., and Moreno, C. 2001. Escaped salmon in the inner seas, southern Chile: facing ecological and social conflicts. Ecological Applications **11**: 1750–1762.

United Nations. 2006. Transfer of technology for successful integration into the global economy; A case study of the salmon industry in Chile. *In* United Nations conference on trade and development. United Nations.

Welcomme, R.L. 1988. International introductions of inland aquatic species. FAO Fisheries Technical Paper **294**.

Table S2: Genetic diversity in Patagonian populations, putative North American founding populations, and overall North American baseline populations.

Population	Reporting group (North America)	N	H_S	*AR*	F_{IS}
**Patagonian populations**					
Baker		24	0.851	9.683	0.039
Aysén		24	0.826	9.450	0.011
Petrohué		24	0.854	10.410	-0.008
Toltén		15	0.804	7.128	0.009
	Mean	21.75	0.834	9.168	0.012
	SD	4.500	0.024	1.420	0.013
**Putative source populations**					
Skagit River upper	Whidbey Basin	55	0.869	10.700	-0.031
Cascade River upper	Whidbey Basin	47	0.877	10.674	0.007
NF Stillaguamish Hatchery	Whidbey Basin	350	0.876	10.927	0.006
Suiattle River	Whidbey Basin	154	0.872	10.791	0.008
Sauk River	Whidbey Basin	115	0.871	11.037	0.003
UW Hatchery su/fa	S Puget Sound fa	140	0.811	9.316	-0.001
Soos Hatchery	S Puget Sound fa	184	0.815	9.988	-0.006
S Prairie Creek	S Puget Sound fa	104	0.805	10.006	0.014
Voights Hatchery	S Puget Sound fa	95	0.814	10.318	0.007
Clear Creek Hatchery	S Puget Sound fa	141	0.809	10.018	0.001
Methow River	Interior Columbia Basin su/fa	143	0.868	11.399	-0.009
Wells Hatchery	Interior Columbia Basin su/fa	144	0.869	11.259	-0.029
Wenatchee River su/fa	Interior Columbia Basin su/fa	135	0.863	11.190	0.009
Hanford Reach	Interior Columbia Basin su/fa	273	0.886	11.974	0.008
Lyons Ferry Hatchery	Interior Columbia Basin su/fa	186	0.870	11.160	0.017
Deschutes River lower	Interior Columbia Basin su/fa	143	0.872	11.345	0.020
Deschutes River upper	Interior Columbia Basin su/fa	144	0.861	10.648	0.005

N_Santiam Hatchery	Willamette R sp	143	0.814	9.732	-0.005
McKenzie Hatchery	Willamette R sp	142	0.817	9.589	-0.001
Lewis River fa	W Cascade fa	93	0.882	11.360	0.006
Sandy River	W Cascade fa	123	0.895	11.839	-0.006
Cowlitz Hatchery fa	W Cascade fa	138	0.873	11.187	0.000
Green River fa	W Cascade fa	55	0.880	11.418	0.031
Cowlitz Hatchery sp	W Cascade sp	139	0.854	10.679	0.003
Kalama Hatchery sp	W Cascade sp	143	0.863	10.844	0.016
Lewis Hatchery sp	W Cascade sp	143	0.868	10.934	-0.015
Necanicum Hatchery	N Oregon Coast	77	0.846	9.639	0.041
Nehalem River	N Oregon Coast	150	0.811	8.803	0.014
Wilson River	N Oregon Coast	137	0.866	10.375	0.003
Kilchis River	N Oregon Coast	58	0.866	10.262	0.015
Trask River	N Oregon Coast	160	0.873	10.524	0.011
Nestucca Hatchery	N Oregon Coast	130	0.858	9.935	0.023
Salmon River fa	N Oregon Coast	102	0.878	10.535	0.021
Siletz River	N Oregon Coast	163	0.882	10.702	0.010
Yaquina River	N Oregon Coast	136	0.868	10.294	0.029
Alsea River	N Oregon Coast	161	0.865	10.246	0.026
Siuslaw River	N Oregon Coast	152	0.887	11.233	0.043
	Mean	137.784	0.858	10.618	0.008
	SD	55.123	0.026	0.703	0.004
**All North American baseline populations**					
	Mean	133.733	0.833	9.810	0.008
	SD	46.405	0.038	1.131	0.002

Table S3: Genetic ancestral contribution of North American lineages to Patagonian Chinook salmon based on population-level CML mixture analysis.

			Patagonian watershed				
ID	Reporting Group	NA Population	Toltén	Petrohué	Aysén	Baker	Pooled
1	Central Valley fa	Stanislaus R		4.1 (1)			1.2 (1)
1	Central Valley fa	Tuolumne R		4.1 (1)			1.2 (1)
5	Klamath R	Klamath R fa	6.9 (1)				1.3 (1)
6	Chetco R	Chetco R	7.3 (1)				1.3 (1)
9	Willamette R sp	N Santiam H			4 (1)	3.3 (1)	1.9 (2)
9	Willamette R sp	McKenzie H			1.3 (0)	15.0 (4)	4.6 (4)
13	N Oregon Coast	Salmon R f	0.6 (0)	4.1 (1)			1.3 (1)
13	N Oregon Coast	Siuslaw R	2.9 (0)	4.2 (1)		0.5 (0)	1.9 (1)
13	N Oregon Coast	Trask R	5.9 (1)	0.1 (0)			1.1 (1)
15	W Cascade fa	Cowlitz H fa	8.9 (2)	3.2 (1)	16.8 (3)	5.4 (1)	8.2 (7)
15	W Cascade fa	Sandy R	0.6 (0)	0.6 (0)	0.5 (0)	5.5 (2)	2.0 (2)
16	W Cascade sp	Kalama H sp	3.0 (0)	15.2 (4)	26.9 (7)	28.7 (7)	19.7 (18)
16	W Cascade sp	Cowlitz H sp	4.3 (1)	6.4 (1)	44.4 (8)	35.8 (8)	23.7 (18)
17	Interior Columbia Basin su/fa	Wenatchee R s/f	6.8 (1)	0.4 (0)			1.4 (1)
17	Interior Columbia Basin su/fa	Hanford Reach	6.2 (1)	8.5 (2)	4.4 (1)	5.5 (1)	6.2 (5)
17	Interior Columbia Basin su/fa	Lyons Ferry H	6.0 (1)	0.1 (0)		0.1 (0)	1.1 (1)
19	S Puget Sound fa	Clear Cr H	0.1 (0)	2.1 (0)	1.3 (0)		0.9 (0)
19	S Puget Sound fa	Soos H		16.9 (5)		0.1 (0)	4.9 (5)
19	S Puget Sound fa	S Prairie Cr	0.1 (0)	1.0 (0)			0.3 (0)
22	Washington Coast	Sol Duc H		4.0 (1)	0.1 (0)		1.2 (1)
22	Washington Coast	Forks Cr H	6.5 (1)	4.0 (1)	0.1 (0)		2.4 (2)
23	Straits of Juan de Fuca	Elwha R	0.2 (0)	5.7 (1)			1.7 (1)
24	Whidbey Basin	Suiattle R		4.2 (1)			1.2 (1)
24	Whidbey Basin	Cascade R U	20.1 (3)	0.3 (0)	0.2 (0)		3.8 (3)
26	E Vancouver Is	Big Qual H	6.3 (1)	2.2 (1)			1.8 (2)
31	S Thompson R	L Adams H	6.4 (1)	0.1 (0)			1.2 (1)
38	SSE Alaska	Clear Cr	0.6 (0)	4.4 (1)			1.4 (1)
39	Nass R	Kincolith R		3.9 (1)			1.1 (1)
	No. Individuals in mixture		(15)	(24)	(20)	(24)	(83)

Notes: Values represent average percent genetic contribution; in brackets, frequency of individual assignments to baseline populations, as inferred from individual's highest assignment probability. Identifiers (ID) correspond to those in Figure 1 (main article).

Figure S1: Confusion matrix of leave-one-out, self assignment test of individual genotypes after conditional maximum likelihood (CML) mixture analysis of North American samples. Values, and colour saturation, correspond to proportion of assignments. Samples (11800 fish genotypes, from 146 populations) were pooled by lineage (45 reporting groups), and sorted by decreasing latitude for display. Note how samples assign to their lineages with high accuracy and rarely to unrelated (distant) lineages, as observed in some simulated, admixed individuals (main article).

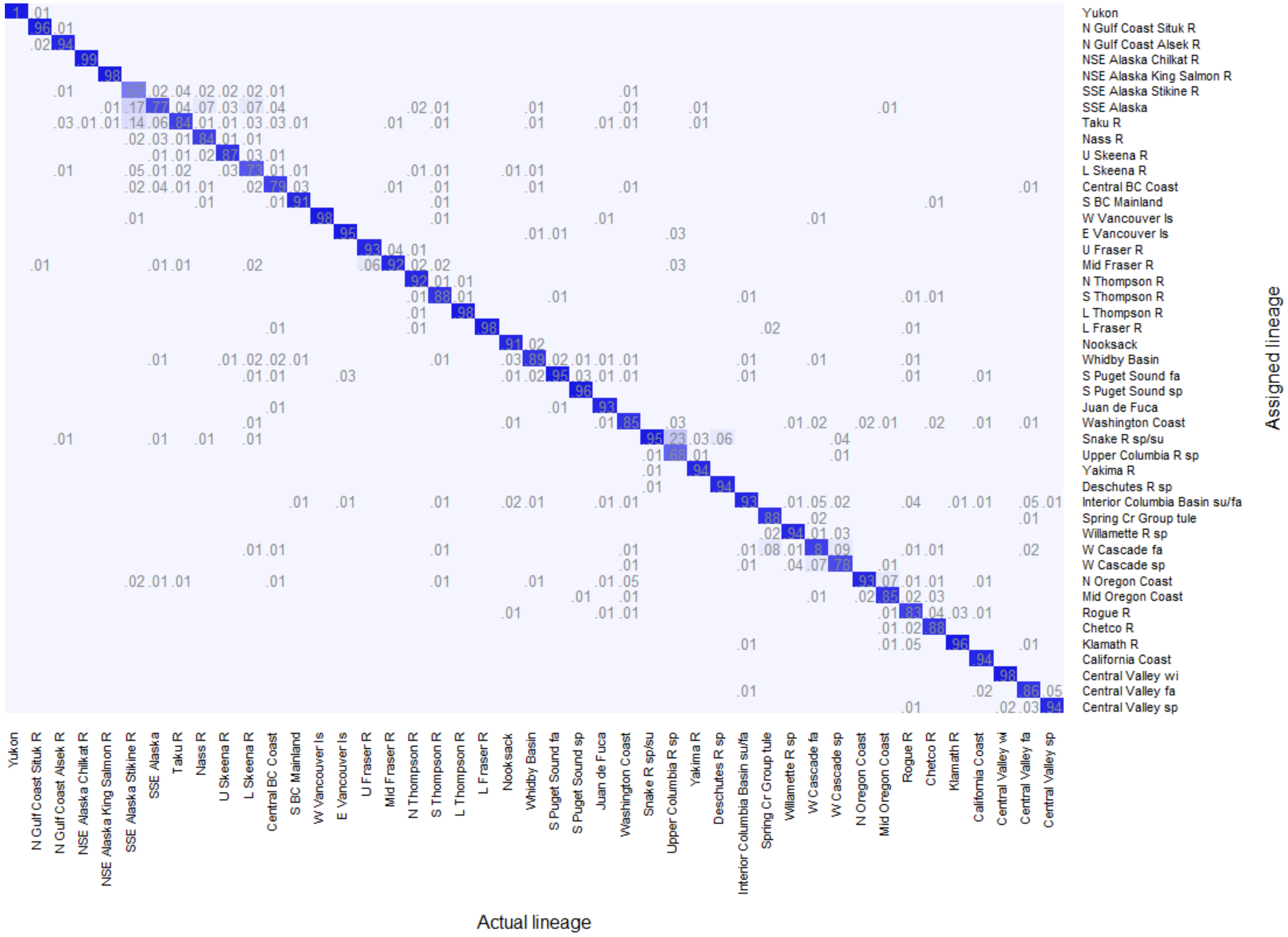

Figure S2: Frequency distribution of individual maximum assignment probabilities from population and reporting group-level CML mixture analysis of Patagonian samples.

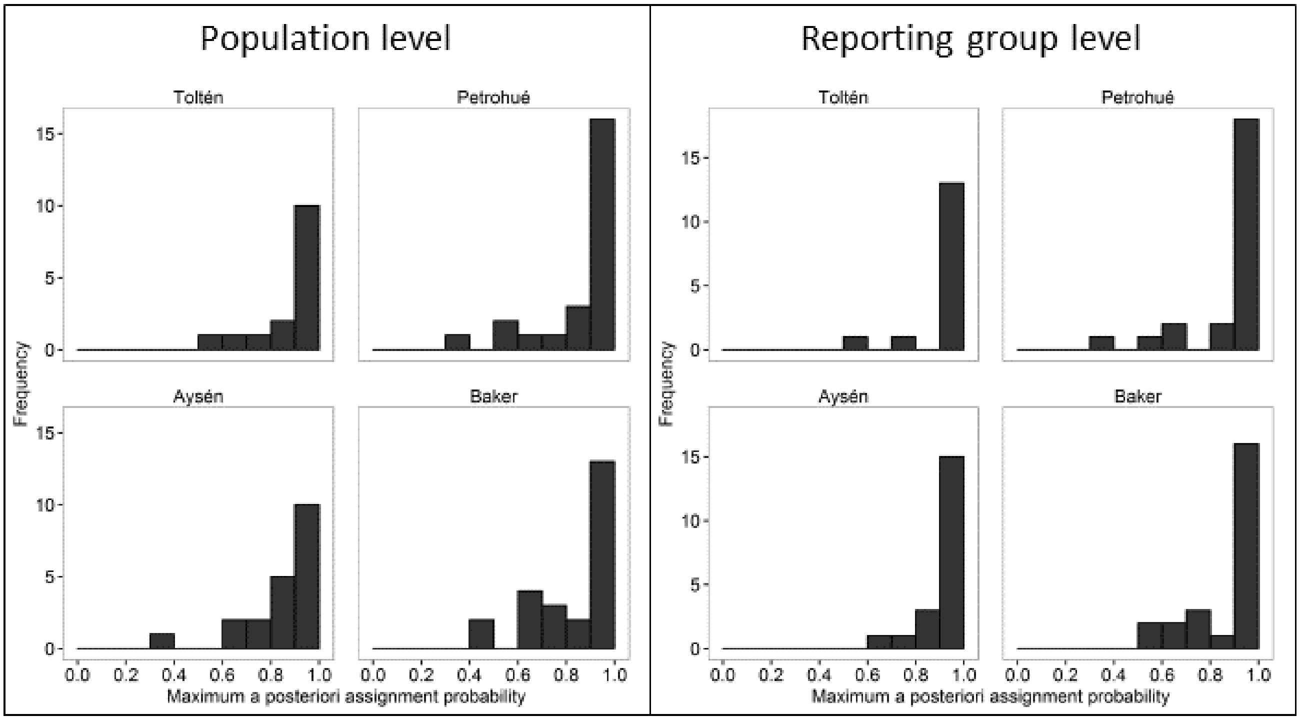

Figure S3: Conditional maximum likelihood (CML) mixture analysis of the simulated mixed-origin Chinook salmon population derived from Cowlitz River Hatchery spring run in the West Cascade spring-run reporting group and Soos Creek Hatchery fall run in the South Puget Sound fall-run reporting group. Distribution of maximum assignment probabilities (a), average percent genetic contribution of reporting groups to the simulated population (b), and individual alternative assignments based on best and second-best assignment probabilities (c). Equivocal assignments [i.e., low assignment probability, symbolized with darker lines in (c)] typically split probabilities between founder lineages, or between founder lineages and genetically similar reporting groups. A small fraction of simulated individuals assigned to unrelated lineages, even with high assignment probabilities in some cases. Reporting groups with no assignments were omitted. Reporting groups were ordered by decreasing order of estimated contribution (b) or increasing latitude (c).

(a)

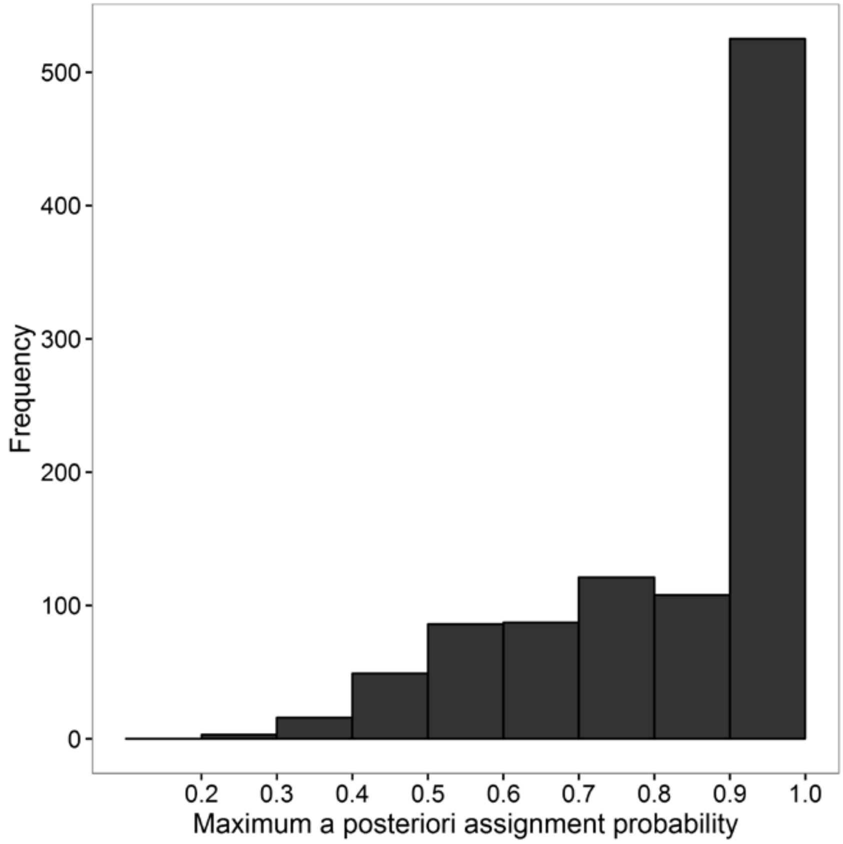

(b)

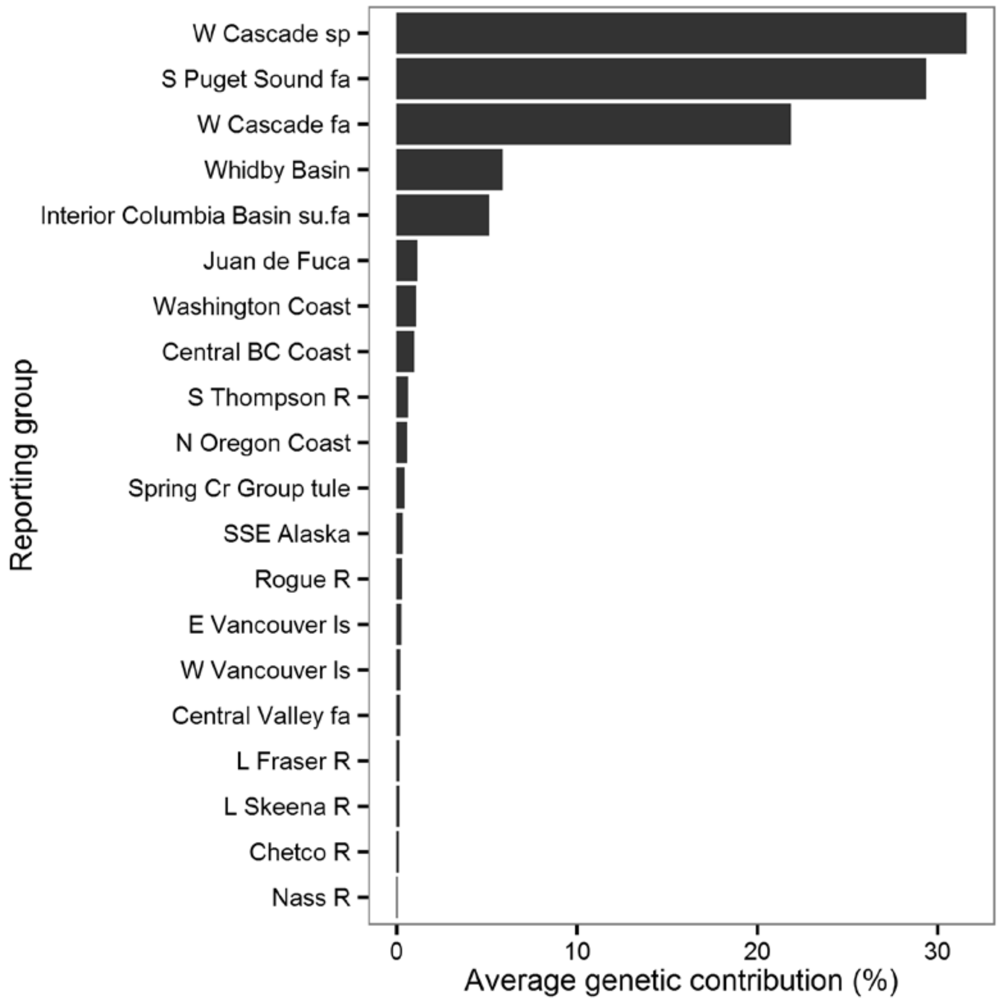
W Cascade sp
S Puget Sound fa
W Cascade fa
Whidby Basin
Interior Columbia Basin su.fa
Juan de Fuca
Washington Coast
Central BC Coast
S Thompson R
N Oregon Coast
Spring Cr Group tule
SSE Alaska
Rogue R
E Vancouver Is
W Vancouver Is
Central Valley fa
L Fraser R
L Skeena R
Chetco R
Nass R
Reporting group
0
10
20
30
Average genetic contribution (%)

(c)

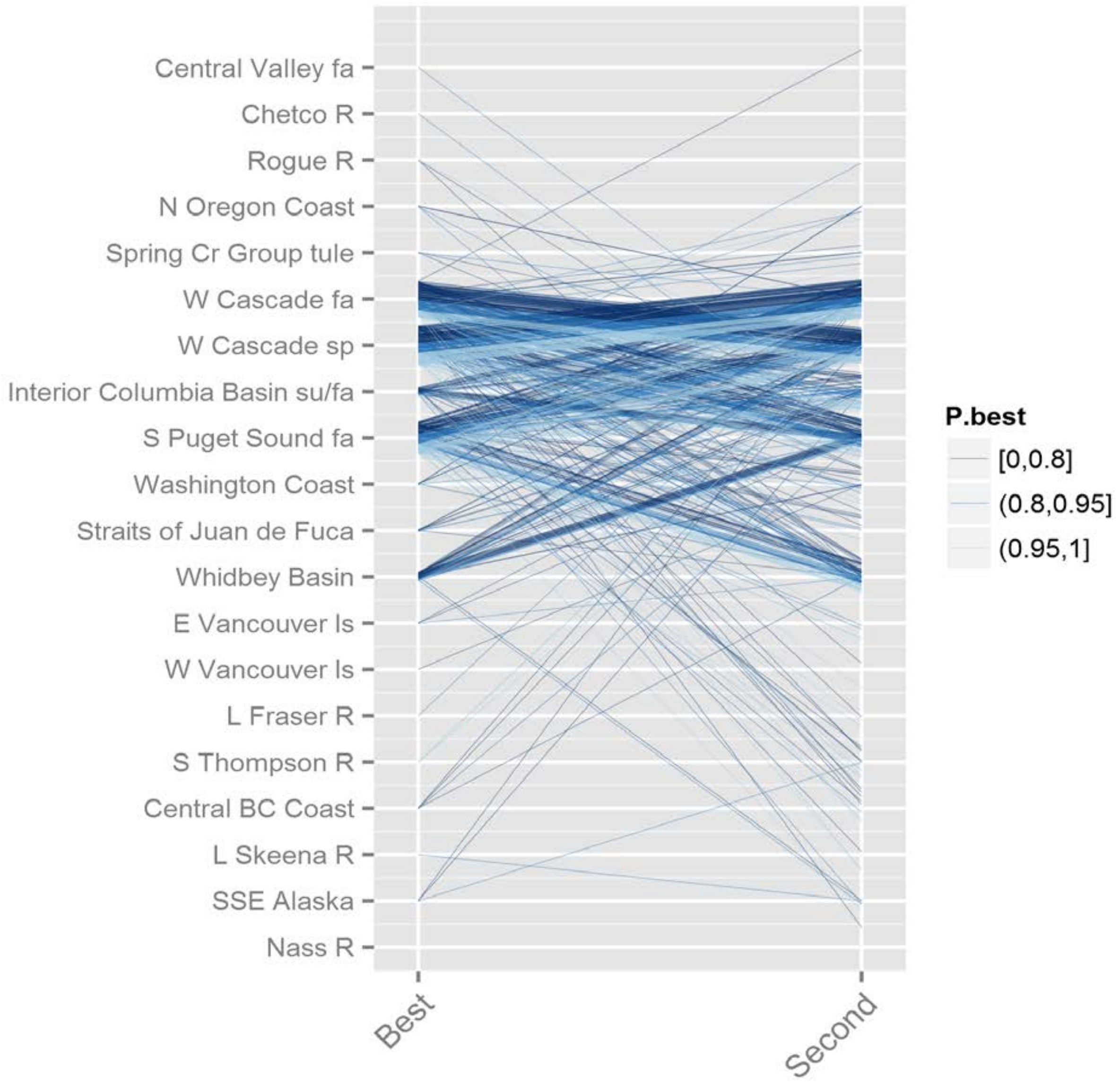
Central Valley fa
Chetco R
Rogue R
N Oregon Coast
Spring Cr Group tule
W Cascade fa
W Cascade sp
Interior Columbia Basin su/fa
S Puget Sound fa
Washington Coast
Straits of Juan de Fuca
Whidbey Basin
E Vancouver Is
W Vancouver Is
L Fraser R
S Thompson R
Central BC Coast
L Skeena R
SSE Alaska
Nass R
Best
Second
P.best
[0,0.8]
(0.8,0.95]
(0.95,1]

Figure S4: Delta-*K* plot to determine appropriate number of groups in model-based clustering present in 8,228 fish from 31 populations distributed among seven North American lineages that potentially contributed founders of Patagonian populations.

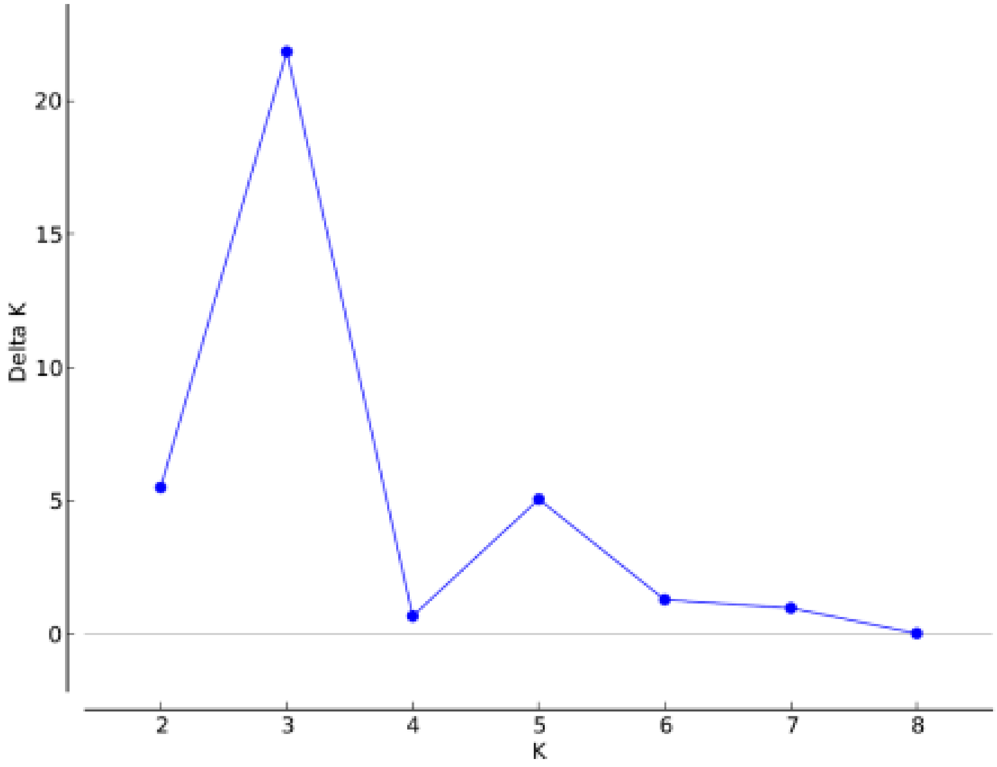

Figure S5: Individual stacked bar plot (STRUCTURE plot) of (a) North American baseline data-set of 8,228 fish from 31 populations distributed among seven North American lineages, and (b) Patagonian samples treated as having unknown origin in the analysis, and plotted by watershed (n = 81). The number of groups was set to *K* = 5, and estimated individual group membership probabilities is shown in colours.

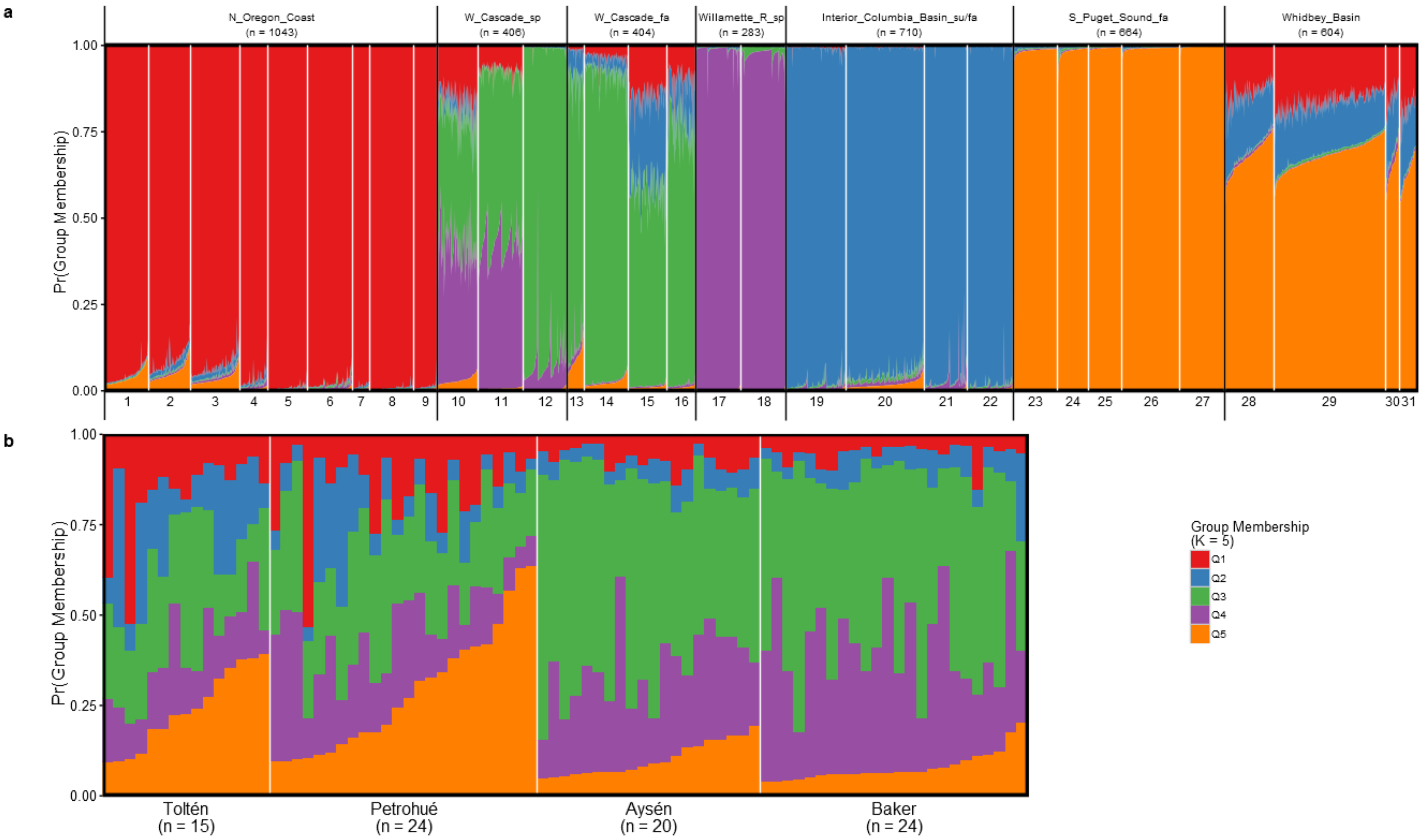

Supplement: Supplementary file 1 — Supplementary Information [file 41598_2017_14465_MOESM1_ESM.pdf]
